# Dialysis and Pregnancy in End Stage Kidney Disease Associated with Lupus Nephritis

**DOI:** 10.1155/2013/923581

**Published:** 2013-11-20

**Authors:** Goutham Sivasuthan, Rumbi Dahwa, George T. John, Dwarakanathan Ranganathan

**Affiliations:** ^1^School of Medicine, The University of Queensland, Brisbane, QLD 4027, Australia; ^2^Department of Nephrology, Royal Brisbane and Women's Hospital, Queensland Health, Butterfield Street, Herston, QLD 4029, Australia

## Abstract

Female patients with systemic lupus erythematosus are often of childbearing age at diagnosis, and though fertility in these patients is similar to the general population, successful pregnancy remains a rare occurrence. This incidence is, however, increasing and the management of these high risk pregnancies is often further complicated by the patient's need for dialysis as a result of lupus nephritis (LN). We share our experience in managing two LN patients with successful pregnancies, one on automated peritoneal dialysis and the other on haemodialysis, as well as a review of cases in the literature.

## 1. Introduction

Pregnancy is an uncommon event in patients with end stage kidney disease (ESKD). According to current Australian statistics from the Australian and New Zealand Dialysis and Transplant (ANZDATA) registry, there have been 105 reported cases of pregnancy in dialysis patients from 1973 to 2009 [1]. Successful pregnancy in a patient with lupus nephritis (LN) and on dialysis is an uncommon occurrence, and this presents a challenge not only to the nephrologist but also to the other medical specialities involved in the care of the patient. 

We present two cases of pregnancy with LN on dialysis from our centre. The first is a rare case of a patient with LN initially on automated peritoneal dialysis (APD) during pregnancy. Our case is the first report in Australia of a successful pregnancy in a patient on APD for ESKD due to LN. 

The second case is of a patient who had ESKD due to LN who required haemodialysis (HD) during her pregnancy. Both pregnancies resulted in live births which continue to thrive. Cases of pregnancy in patients with LN on dialysis are still uncommon occurrences, and we have reviewed the cases reported in the literature, along with our own centre's experience, in managing these patients. 

## 2. Case One

A 30-year-old female with ESKD on APD who at the time of her pregnancy was a gravida 4, para 1, and abortus 2 was admitted to our hospital. She had a 14-year-old son born by spontaneous vaginal delivery a year before she was diagnosed with systemic lupus erythematosus (SLE), and subsequently she has had two medical terminations of pregnancy due to SLE flares. SLE was diagnosed at age 17, and over the years it had manifested with arthralgia, episcleritis, seizures, and a biopsy proven WHO Class IV lupus nephritis and advanced ESKD. She had started APD at 28 years of age. 

At 9 weeks of gestation, she was transferred to our tertiary institution from one of the peripheral hospitals in order to plan coordinated care of her pregnancy with nephrologists, obstetric physicians, haematologists, obstetricians, and rheumatologists. 

At the initial pregnancy assessment, her blood pressure (BP) was 95/60 mm Hg with a symptomatic postural drop of 25 mm Hg. Because of this, her prepregnancy regimen of one 6-litre bag of 1.5% and two bags of 2.5% over 8 hours with 6 cycles was changed to three 1.5% 6-litre bags. She had no clinical signs of lupus activity, and laboratory investigations showed a haemoglobin of 96 g/L, platelet count of 319, erythrocyte sedimentation rate 122 mm/hr, C3 0.08 g/L, C4 0.67 g/L, anti-double-stranded DNA antibody (dsDNA) 28 IU/mL, positive direct Coombs' test, and low haptoglobin 0.19 g/L but normal reticulocyte count, lactose dehydrogenase (LDH), and bilirubin. She was started on plaquenil and prednisolone for microangiopathic haemolytic anaemia (MAHA). 

The patient was counselled about the risks both to her and foetus, of continuing with the pregnancy, and she made an informed decision to proceed with the pregnancy. She was started on low dose aspirin and daily enoxaparin for preeclampsia prophylaxis. During pregnancy, the dose of erythropoietin stimulating agent (ESA) was titrated according to haemoglobin.

The PD regimen was modified throughout her pregnancy with the aim of keeping blood urea at or less than 15 mmol/L. At 20 of weeks gestation, urea increased, and she was switched to continuous ambulatory peritoneal dialysis (CAPD) to allow for better variance of her prescription. The total dose of PD was five exchanges with 1.5% bags with volumes of 1.5 litres.

At 31 weeks of gestation, the patient developed preeclampsia manifested by blood pressure of 180/110 mm Hg, headache, and epigastric pain. A 900 g male baby was delivered by emergency transperitoneal lower segment caesarean section with Apgar scores 7 and 9 at 1 and 5 minutes, respectively. The baby was admitted to a special care nursery where he required supplemental oxygen but was otherwise well. 

 The patient started on HD via a central venous dialysis catheter at day 2 postpartum. She was discharged on day 5 postpartum and continued on HD until day 20 when she restarted PD. Throughout her pregnancy and in the postpartum period, no other features of lupus activity manifested apart from the MAHA.

## 3. Case Two

The second case is of a 20-year-old female who was gravida 1, para 0, and abortus 0 diagnosed with SLE at the age of 15. She presented initially with arthralgia and subsequently developed biopsy proven WHO class IV LN at the age of 18. She was commenced on steroids and mycophenolate mofetil, but renal dysfunction progressed to ESKD due to nonadherence with therapy. She also developed significant hypertension which was treated with amlodipine, perindopril, and candesartan.

Her pregnancy was diagnosed at 6 weeks of gestation. She made an informed decision to proceed with the pregnancy. 

 At initial assessment, there were no clinical signs of lupus activity. Her BP was 160/99 mm Hg, and laboratory investigations showed a urea of 17.9 mmol/L, creatinine 253 *μ*M/L, estimated glomerular filtration rate (eGFR) 21, haemoglobin of 89 g/L with normal iron studies, vitamin B12 and red cell folate, C3 0.94 g/L, C4 0.23 g/L, dsDNA 8 IU/mL, negative direct Coomb's test, normal reticulocyte count, LDH, haptoglobolin, and bilirubin. The antihypertensive medications were changed to methyldopa and labetalol. Low dose aspirin and enoxaparin were given daily for prophylaxis of preeclampsia. She was treated with an ESA to maintain haemoglobin at 110–120 g/dL. 

Throughout the pregnancy, she continued to have elevated blood pressures with systolic blood pressures up to 200 mm Hg which resulted in multiple admissions, and her medications included hydralazine and nifedipine. 

At 8 weeks of gestation, the patient was started on pre-emptive HD with an aim to keep urea below 15 mmol/L. The laboratory investigations at the time of dialysis initiation showed a urea of 16.6 mmol/L, creatinine of 274 umol/L, and a decline in eGFR to 19. Her dialysis prescription initially was for 6 times a week, 3 hours using a F8 dialyser with a blood flow rate (BFR) of 200 mL/minute and of dialysate flow rate 300 mL/minute. Dry weight was assessed at each dialysis, and the aim was for her to have an approximate weight gain of 0.25 kg/week till 20 weeks of gestation. In order to maintain urea below 15 mmol/L, dialysis duration was increased to 4 hours, and BFR and dialysate flow rate were increased to 250 mL/min and 500 mL/min, respectively, at 22 weeks of gestation. 

She was admitted to hospital at 29 weeks of gestation following a two-day history of feeling unwell and intermittent nausea. Cardiotocography on admission showed poor variability. Fetal ultrasounds scan showed intrauterine growth restriction (IUGR) with an estimated fetal weight of 975 grams which was below the third centile. She received steroid prophylaxis. The patient had an emergency lower segment caesarean section with the birth of a 906 g live male infant with Apgar scores of 1, 2, and 8 at 1, 5, and 10 minutes. The baby was admitted to the special care nursery where he continued to progress well. 

## 4. Case Discussion

SLE predominantly affects women of childbearing age and therefore pregnancy in SLE is a significant concern. Fertility in patients with SLE is similar to the general population though pregnancy in patients with ESKD and patients on dialysis is uncommon [2]. The incidence in Australia from 1973 to 2009 is 0.6% [1]. Contraceptive use is encouraged in these patients to ensure that pregnancy does not occur during a period of lupus activity, preferably delaying conception until 6 months of quiescence [3]. The advice can be largely attributed to the three-fold increased risk of stillbirth for pregnancies with active SLE activity, despite insignificant increase in miscarriages [4]. The estimated pregnancy rate is likely to be higher as the number of abortions is unknown and not always included. The low pregnancy rate is due to reduced fertility caused by changes in hormones regulating reproductive function which results in anovulation. It has also been linked to reduced libido in ESKD patients.

The first report of a successful pregnancy in a chronic HD patient was made by Confortini et al. in the early 1970s [5]. Pregnancy in patients with ESKD is associated with both fetal and maternal adverse outcomes, and in the past most clinicians discouraged pregnancy in patients with ESKD. Maternal risks include hypertension whilst fetal risks include IUGR and prematurity. A rise in fertility rates has been noted as dialysis care has improved. An increase in the number of chronic dialysis patients who have menstrual periods has been noted, with Holley et al. finding a rate of 50% [6]. In the late 1970s Perez et al. had reported the incidence as 10% [7]. It is known however that not all menstrual cycles are associated with ovulation. Patients with residual renal function are more likely to ovulate, and thus pregnancy is more likely to occur during the early years of dialysis. Both of our patients still had residual renal function and both patients made 700–800 mL urine/day. Wing et al. report a series of pregnancies occurring after an average length of dialysis of 2.2 years, and Giatras et al. noted that 47% of pregnancies reported occurred during the first two years on dialysis [8, 9]. There are case reports of patients falling pregnant after being on dialysis for longer with Hsieh et al. reporting three cases of pregnancy in women who had been on dialysis for more than five years [10]. A correlation between an increased time on dialysis during pregnancy and improved outcomes during pregnancy has been observed [11]. 

The incidence of pregnancy is two to three times more common in HD patients compared with PD patients [11]. It is unclear whether the difference is due to an effect of PD itself or due to differences in hormonal milieu. 

Of the documented 105 pregnancies in a 36-year period for Australian ESKD patients on dialysis, 48% of the pregnancies resulted in a live delivery. Seven of these pregnancies were in patients with SLE, and of these only one had a live delivery [1]. This illustrates the poor outcomes that are associated with pregnancy in SLE patients. Severe hypertension is the most life-threatening complication of pregnancy in ESKD patients [12]. These complications are more severe in patients with SLE if there is evidence of disease activity at the time of conception or early in the pregnancy [4]. These complications highlight reasons for the rare occurrences of successful pregnancies on dialysis as a result of SLE, and Table 1 summarises the cases present in the literature.

## 5. Management of Pregnancy

For antenatal management, Dudley and Branch have suggested fortnightly visits for the first and second trimester with weekly visits in the third trimester [13]. 

Once pregnancy has been confirmed, it is important to review the patient's current medications in regard to safety in their use during pregnancy. Tables 2(a) and 2(b) list commonly used drugs in dialysis and SLE, respectively, along with current safety ratings by Australian Therapeutic Goods Administration [14]; Table 2(c) lists the key for these ratings [15].

There is variation on whether pregnancy increases the risk of flares with some authors quoting an increased risk of a flare of LN during pregnancy,while others report no increased risk of flares when compared with nonpregnant patients [16–18]. Flares can occur at any time during the pregnancy or in the postpartum period. The frequency of flares is higher in patients with active lupus at conception and also in those with a positive lupus anticoagulant. In our patient, this manifested as MAHA which was controlled with plaquenil and steroids. Measurement of complement levels is one of the parameters assessed for lupus activity, and this normally manifests with reduced complement levels. In pregnancy, however, the complement levels can increase up to 50% from increased synthesis, and thus when assessing for disease activity, a downward trend or a low normal level may still indicate disease activity. 

Patients on PD are less likely to achieve pregnancy than HD [19]. Possible causes for this difference may be a result of the hypertonic dialysate in the peritoneum or from prior occurrences of peritonitis causing adhesions and failure of implantation [11]. Our case one was already on APD, and this was continued during the pregnancy with change to CAPD in the latter stages of pregnancy. There is very little in the literature in terms of guidelines for PD in pregnancy. The potential advantages of PD are that it allows for continuous dialysis and less changes in the intravascular maternal volumes when compared with HD.

The aim of increasing dialysis in pregnancy is to provide a less azotemic uterine environment. Holley et al. suggest starting dialysis at a 28.6 mmol/L urea and aiming to keep it below 18 mmol/L, while Jefferys and colleagues recommend keeping the urea less than 10 mmol/L and the creatinine as low as possible [6, 20]. With both our patients, we aimed to keep the urea less than 15 mmol/L. The increased dialysis delivery also allows for lifting of dietary restrictions and results in less hypotensive episodes in HD patients. Dialysis times in HD patients are generally increased to greater than 20 hours per week.

Increasing dialysis may also be beneficial in reducing polyhydramnios risk as this is thought to be caused by urea diuresis in the foetus. In a case series of 5 patients with ESRD managed with CAPD during their pregnancies, none of them developed polyhydramnios and the authors suggest that this was due dialysis being initiated preemptively [20].

The dialysate bicarbonate composition may need alteration due to the more frequent dialysis and the normal metabolic changes that occur in pregnancy. Respiratory alkalosis occurs in normal pregnancy and is compensated for by metabolic acidosis, resulting in a serum bicarbonate level of 18–20 mmmol/L. Hou et al. thus recommend the reduction of dialysate bicarbonate in order to prevent the flux of bicarbonate from dialysate given the increased frequency of dialysis [21].

Anaemia is common in normal pregnancy, and pregnancy is known to cause resistance to erythropoietin. This is thought to be mediated by cytokines whose production is increased in pregnancy. In the case of our first patient, haemolytic anaemia also contributed as did HD in the second case. Both patients were managed with an ESA titration to target haemoglobin of 110–120 g/L.

Hypertension management is critical, and in pregnant dialysis patients, in addition to monitoring for preeclampsia, assessment of fluid status is vital as the raised blood pressure may be due to fluid accumulation. It is particularly important in pregnant patients to carefully remove fluid as hypotension may result in uterine hypoperfusion.Both our patients also had an increased risk of preeclampsia due to SLE (30–50%) as compared with an incidence of 6–10% in the general population [13, 22]. The use of low dose aspirin and heparin is a well-known method of reducing risk of preeclampsia [23, 24]. The presence of the antiphospholipid antibody further increases risk of hypertension as this antibody predisposes to arterial hypertension by causing endothelial damage. 

Making the distinction between a flare of LN and preeclampsia can be difficult as both conditions can present with hypertension, peripheral oedema, and proteinuria. Severe cases of HELLP syndrome can also manifest with MAHA. Differentiating the two conditions requires both clinical and laboratory measurements. Lupus flares are more likely to be present if accompanied by hypocomplementemia, high or rising anti-dsDNA antibody titre, and active urinary sediment. It is important to make the distinction as the treatment modalities are entirely different.

Caesarean section can be transperitoneal or extraperitoneal. Our patient had a transperitoneal caesarean section and restarted peritoneal dialysis at day 20. Hou recommends that peritoneal dialysis can be restarted at 24 hours if caesarean section is done extraperitoneally [25].

Managing the multiple aspects of LN pregnancy with dialysis can be a challenge, and no set guidelines exist for these rare cases.  Table 3 shows the general management guidelines at our centre for such cases.

## 6. Fetal Outcomes

In a review of pregnancies of women on chronic HD from 1992–2003, Holley and Reddy analysed 6 separate reports of pregnancies, and the average percentage of surviving infants was 43% [11]. This is double the percentage previously described in 1980 by the Registration Committee of the European Dialysis and Transplant Association [26].

Hypertension is frequently seen in lupus pregnancy which leads to both prematurity and IUGR [18].

## 7. Conclusion 

Pregnancy in patients with ESRD and in particular in patients with LN is associated with a high fetal and maternal risk. With close maternal and fetal surveillance in conjunction with multidisciplinary management, favourable outcomes are possible in such patients. We report two cases managed by two different modes of dialysis, both resulting in successful outcomes.

## Figures and Tables

**Table 1:** Cases of live births in dialysis patients with systemic lupus erythematous in the literature.

Dialysis modality	Age	Weeks of gestation	Birth weight (g)	Complications	Reference
Peritoneal dialysis	39	36	2338	Preeclampsia	Hou et al. [21]

Peritoneal dialysis	27	39	2480	Haemorrhagic peritoneal drainage fluid, pre-eclampsia	Altay et al. [27]

Haemodialysis	25	32	1400	Hypertension, Diabetes mellitus	Malik et al. [28]

Haemodialysis	26	31	1810	Hypertension	Chou et al. [29]
Peritoneal dialysis	39	35	2388	Preterm	
Peritoneal dialysis	31	34	1004	Intrauterine growth restriction	

Haemodialysis	20	35	1440	Hypertension, fetal distress, anaemia, and haemorrhage	Romão et al. [30]
Haemodialysis	22	27	1030	Fetal distress, anaemia, and haemorrhage	

**Table:** (a)

Drug	Australian category for prescribing medicines in pregnancy [14]
ACE inhibitors	D
Angiotensin II receptor blockers	D
Calcium channel blockers	C
Beta-adrenergic blocking agents	C
Diuretics	
Aldosterone antagonist	B3
Carbonic anhydrase inhibitor	B3
Loop diuretic	C
Potassium-sparing diuretic	C
Thiazide diuretic	C
Thiazide-like diuretic	C
Vasopressin receptor 2 antagonist	D
Phosphate binders	
Lanthanum carbonate	B3
Sevelamer	B3
Erythropoietin	A
Iron	
Iron polymaltose	A
Iron sucrose	B3
Bone disease	
Calcitriol	B3
Paricalcitol	C
Cinacalcet	B3
Itching	
Diphenhydramine	A
Hydroxyzine	A
Cetrizine	B2

**Table:** (b)

Drug	Australian category for prescribing medicines in pregnancy [14]

Hydroxychloroquine	D
Azathioprine	D
Mycophenolate mofetil	D
Cyclophosphamide	D
Cyclosporin	C
Corticosteroids	A
Nonsteroidal anti-inflammatory drugs (NSAIDs)	C

**Table:** (c)

Category	Definition [15]
A	Drugs which have been taken by a large number of pregnant women and women of childbearing age without any proven increase in the frequency of malformations or other direct or indirect harmful effects on the foetus having been observed.

B1	Drugs which have been taken by only a limited number of pregnant women and women of childbearing age, without an increase in the frequency of malformation or other direct or indirect harmful effects on the human foetus having been observed. Studies on animals have not shown evidence of an increased occurrence of fetal damage.

B2	Drugs which have been taken by only a limited number of pregnant women and women of childbearing age, without an increase in the frequency of malformation or other direct or indirect harmful effects on the human foetus having been observed. Studies on animals are inadequate or may be lacking, but available data show no evidence of an increased occurrence of fetal damage.

B3	Drugs which have been taken by only a limited number of pregnant women and women of childbearing age, without an increase in the frequency of malformation or other direct or indirect harmful effects on the human foetus having been observed. Studies on animals have shown evidence of an increased occurrence of fetal damage, the significance of which is considered uncertain in humans.

C	Drugs which, owing to their pharmacological effects, have caused or may be suspected of causing, harmful effects on the human foetus or neonate without causing malformations. These effects may be reversible. Accompanying texts should be consulted for further details.

D	Drugs which have caused, are suspected to have caused, or may be expected to cause an increased incidence of human fetal malformations or irreversible damage. These drugs may also have adverse pharmacological effects. Accompanying texts should be consulted for further details.

X	Drugs which have such a high risk of causing permanent damage to the foetus that they should not be used in pregnancy or when there is a possibility of pregnancy.

**Table 3:** Pregnancy and dialysis monitoring guidelines at the Renal Department of the Royal Brisbane and Women's Hospital.

Guideline	Recommendation/observations/frequency
Dialysis prescription	
*HD *	
Dialyser	High-flux, high-efficiency
Duration and frequency	At least 20 h/week
Blood flow rate	300 mL/min
Dialysate flow rate	500 mL/min
Dialysate composition	Calcium 1.75 mmol/L, bicarbonate 25 mmol/L, and glucose 5 mmol/L
Weight review	Weekly clinically + blood volume monitoring weekly
Fluid status	Prefered to leave “wet” as opposed to dry to avoid hypotension
Anticoagulation	Unfractionated heparin (1500 u bolus and 750 u hourly; off for the last 60 minutes)
Erythropoietin therapy	Recommendation to maintain haemoglobin > 110 g/L. May need higher doses
Iron therapy	Intravenous iron to maintain transferrin saturation > 25%
Vital signs	Each dialysis
Blood pressure parameters	Avoidance of hypotension imperative
ensure that phosphate binders and active vitamin D are adjusted as needed	
Pathology*	Perform full blood counts, electrolyte, and liver function tests weekly. Vitamin B12 checked every 3 months. Check dialysis adequacy using Kt/V ratio weekly. Other bloods as routinely done in dialysis patients
Haemoglobin	Maintain 110–120 g/L
Iron studies	Aim to achieve a transferrin saturation above 25%
Vitamin B12/folate	Suggest supplement folate 5 mg daily
Magnesium	Keep in normal range
Urea	Aim to keep pre-dialysis < 15 mmol/L
Bicarbonate	Keep in normal range before dialysis
Phosphate	When dialysis hours increased it is important to avoid low phosphate
Urate	Monitor levels
Diet*	
High protein	Dietician to review regularly
Supplements	Suggest folate 5 mg daily, vitamin B1 daily, vitamin D 1000 iu daily, and calcitriol (adjust according to phosphate and calcium)
Aspirin	To consider this in consultation with obstetric physicians/obstetricians
Fetal monitoring*	
Ultrasonography	Frequent to monitor growth discussision with obstetricians/obstetric physicians

*Guidelines also apply for peritoneal dialysis.
